# Two-stage total hip arthroplasty for patients with advanced active tuberculosis of the hip

**DOI:** 10.1186/s13018-016-0364-3

**Published:** 2016-03-30

**Authors:** Liangjun Li, Ke Chou, Jianliang Deng, Feng Shen, Zhiyong He, Shuguang Gao, Yusheng Li, Guanghua Lei

**Affiliations:** Department of Orthopedics, Xiangya Hospital, Central South University, Changsha, Hunan China; Department of Orthopedics, Changsha Central Hospital, Changsha, Hunan China

**Keywords:** Total hip arthroplasty, Active tuberculosis, Hip, Two-stage

## Abstract

**Background:**

Treatment of advanced active tuberculosis (TB) of the hip is confronted with great challenges. Although one-stage total hip arthroplasty (THA) is considered as a safe procedure for most patients by some authors, there are still exceptions. The purpose of this paper was to investigate the feasibility and effectiveness of two-stage THA for selected patients with advanced active TB of the hip.

**Methods:**

Nine consecutive patients with advanced active tuberculous arthritis of the hip were reviewed in this study. Out of these nine patients, the hips of five were destroyed extensively with difficulties of thorough debridement at one operation, and the hips of the other four were detected of sinus tracts. Nine patients received the two-stage total hip arthroplasty (THA) protocol and the perioperative antituberculous medication between January 2008 and December 2013. During the first stage, a debridement was carried out after at least 2 weeks of antituberculous chemotherapy to remove abscesses and infected and necrotic tissues as thoroughly as possible, followed by antituberculous chemotherapy for a minimum of 3 months (average 4.2 months). During the second stage, hip prosthesis was implanted if the erythrocyte sedimentation rate (ESR) and the C-reactive protein (CRP) were normal and the wound was well healed. Antituberculous chemotherapy was continued for 6–9 months postoperatively to constitute a total duration of a minimum of 12 months after the first operation. The patients were then evaluated based on the reactivation of infection, the Harris hip score system, X-ray, ESR, and CRP.

**Results:**

The average follow-up was 40 months (range, 18–72 months). No reactivation of TB or superimposed infection was observed in all patients. The ESR and CRP returned to the normal level with no liver injury. The average Harris hip score was increased from 35 (range, 15–55) preoperatively to 91.5 (range, 83–97) at the final follow-up. The X-ray film showed no prosthesis shift or loosening.

**Conclusions:**

Two-stage THA is an alternative treatment option for patients with advanced active tuberculosis of the hip under some difficult conditions. The hip with sinus tracts or destroyed extensively with difficulties of thorough debridement at one operation may be regarded as indications.

## Background

Tuberculosis (TB) has re-emerged as an important medical problem all over the world. There are approximately 30 million people suffering from tuberculosis globally, and 1 to 3 % of them have involvement of the skeletal system [1]. TB of the hip constitutes about 15 % of all patients of osteoarticular TB and is the most frequent site of bone involvement after the spine [2, 3]. As a troublesome disease, it often results in severe cartilage and bone destruction and degeneration of the hip if early diagnosis and treatment was missed [4]. At the advanced stage, treatment of active TB of the hip can be confronted with great challenges.

Traditionally, arthrodesis or Girdlestone’s excision arthroplasty is applied for pain relief and infection control, but the functionality of the hip is unsatisfactory [2, 5]. Total hip arthroplasty (THA) has been operated successfully in patients with quiescent TB [1, 6, 7]. However, THA for the management of active tuberculous arthritis is a controversial treatment option due to the potential risk of reactivation of infections. With a radical debridement and a complete course of antituberculous chemotherapy perioperatively, one-stage THA has been considered as a safe method to treat active tuberculosis of the hip by several authors [5, 8–11], but it requires strict indications and proficient technical skills. If one-stage THA was chosen to treat active hip tuberculosis, complete curettage and debridement of infected tissues at the time of operation would be a crucial procedure to guarantee the success of surgery [12, 13]. Shen et al*.* [10] and Yoon et al. [14] suggest that if a thorough debridement cannot be achieved, a two-stage surgery should be considered. Furthermore, most authors suppose that patients with sinus drainage are not good candidates for one-stage joint arthroplasty [3, 11, 14, 15]. The presence of sinus drainage usually indicates pyogenic superinfections from *S. aureus* or other pathogens. Sinus tracts can also increase the difficulties of thorough debridement. In this study, nine consecutive patients were selected to receive two-stage THA, with the purpose to evaluate our experiences on the feasibility and effectiveness of the treatment.

## Methods

**Fig. 1:**
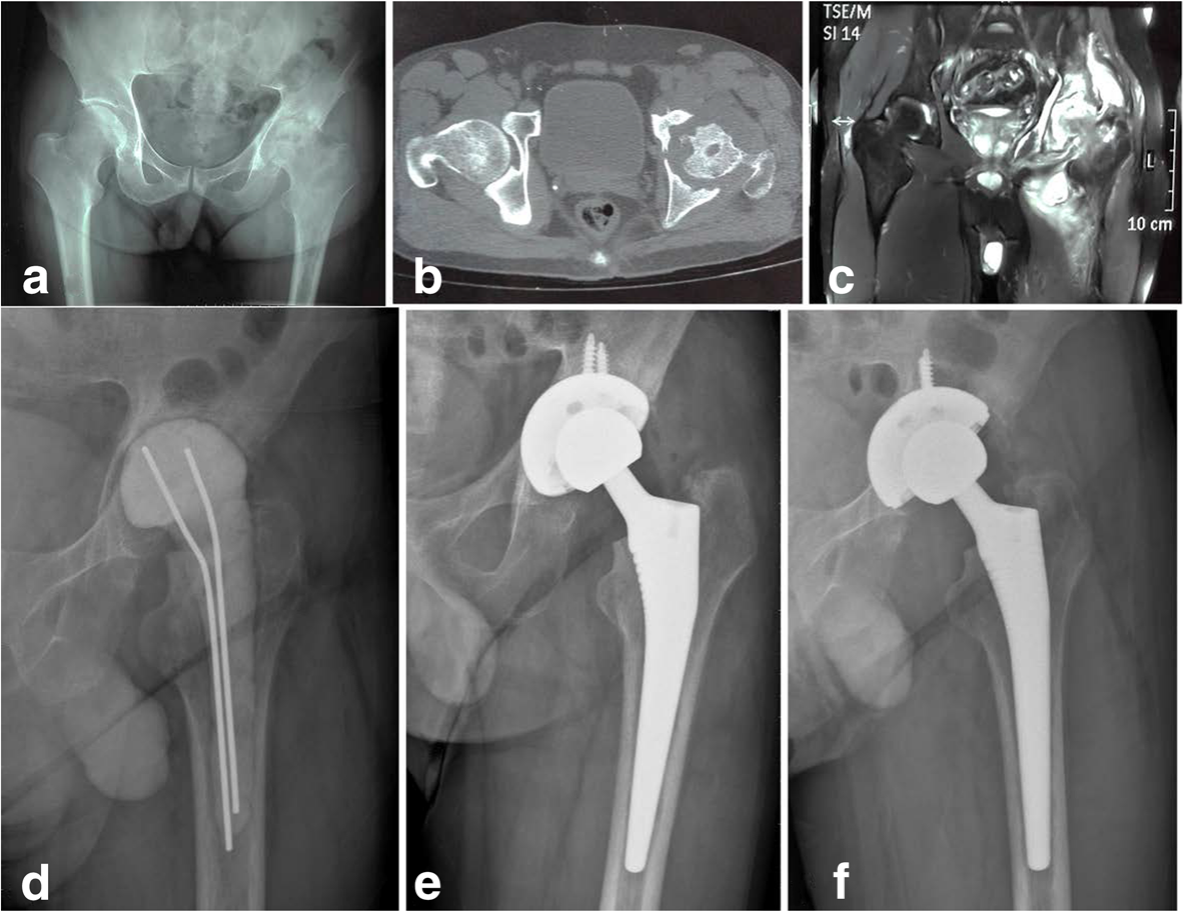
**a** A preoperative radiograph showing bony destruction and narrowing of the left hip joint space. **b** The CT scan showing bony destruction and multiple cavities of the femoral head. **c** The MR scan showing the active disease with extensive soft tissue abscesses. **d** The femoral head was excised during the first operation and a cement spacer was planted in. **e** The radiograph taken 1 week after the second operation. **f** The radiograph of the same hip at 1 year, which shows that the femoral stem and acetabular cup are radiologically stable

**Fig. 2:**
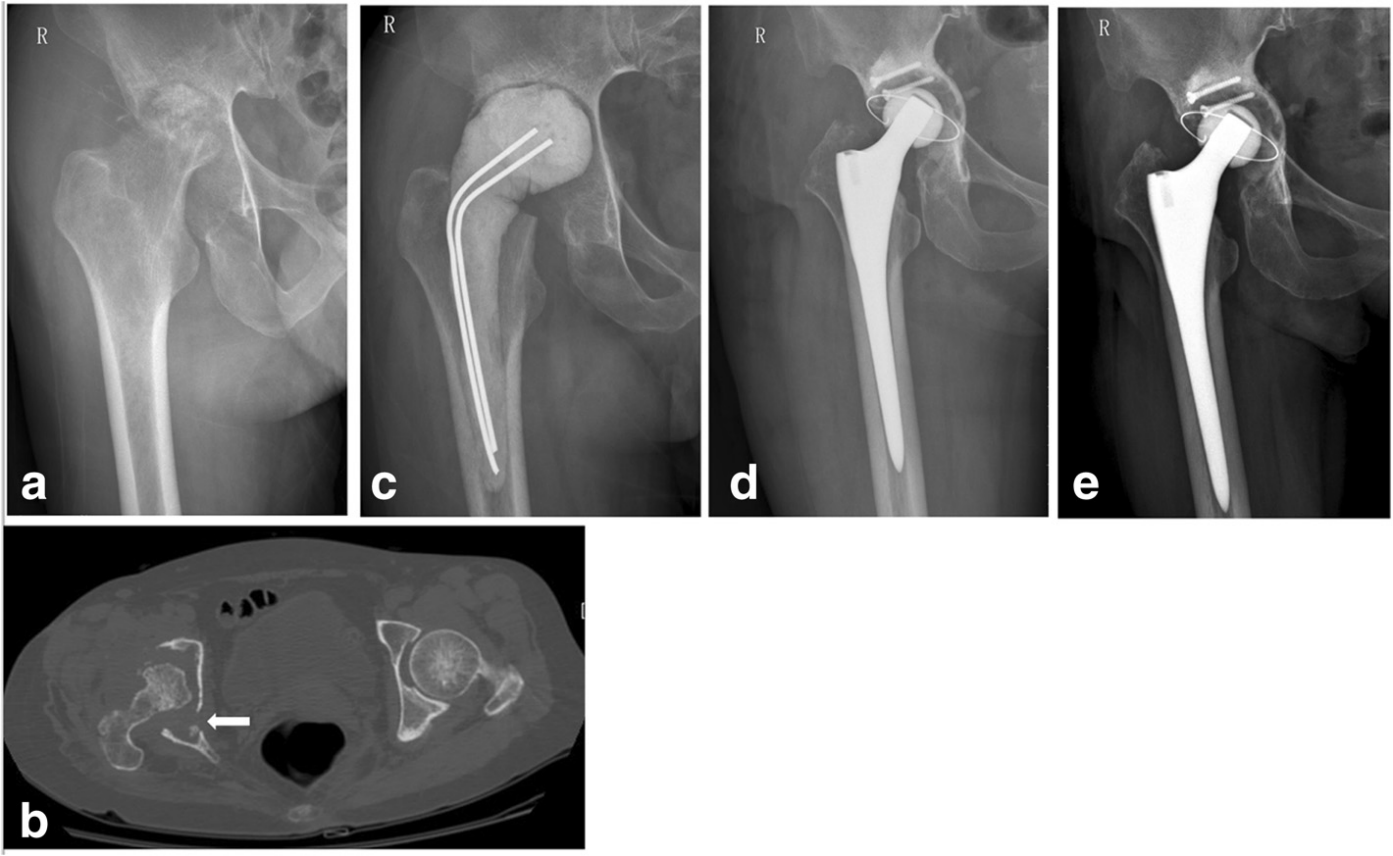
**a** A preoperative radiograph showing bony destruction and narrowing of the right hip joint space. **b** The CT scan showing bony destruction and a sinus tract to the pelvis (*white arrow*). **c** The femoral head was excised, and a cement spacer was planted in during the first operation. **d** The radiograph taken after the second operation showing bone graft fixed with two nails at the acetabular side. **e** The radiograph taken 4 years later showing no evidence of osteolysis or disease reactivation

**Fig. 3:**
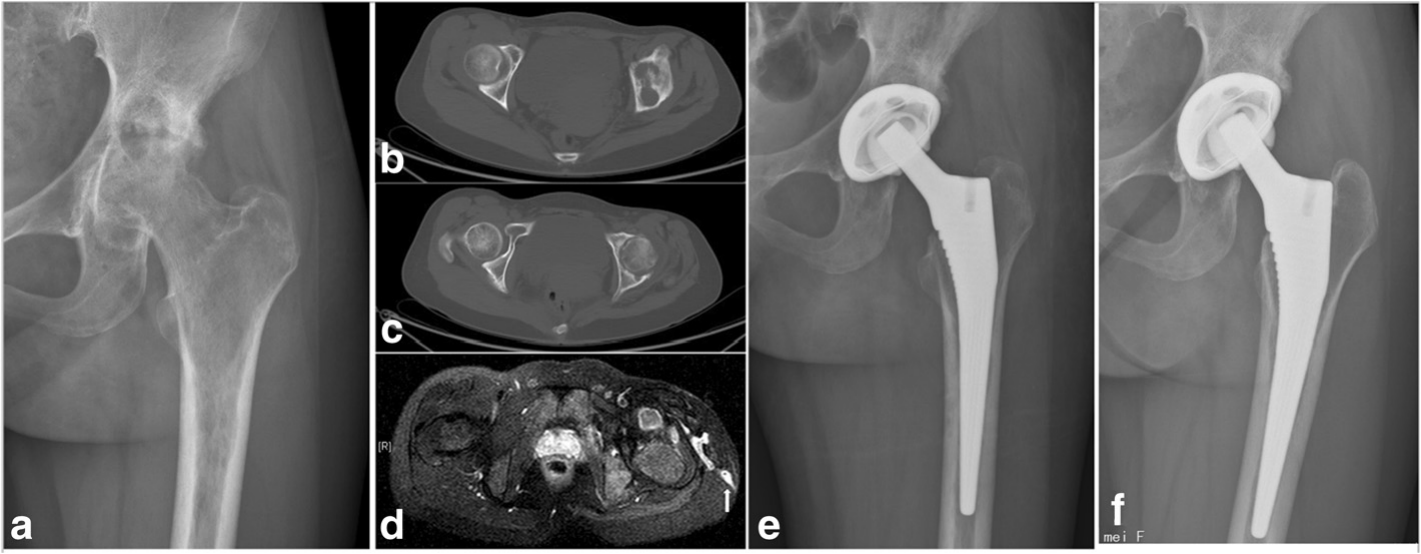
**a** A preoperative radiograph showing bony destruction and narrowing of the left hip joint space. **b** The CT scan showing bony destruction at the acetabular side. **c** The femoral head was relatively intact. **d.** An MR scan showing a sinus to the thigh (*white arrow*). There was no superinfection with this patient. A debridement with the femoral head reserved was carried out at the first operation. **e** The radiograph taken after the second operation, which shows that the bone defect on the top of the acetabulum was filled with allograft bone at the second operation. **f.** The femoral stem and acetabular cup are radiologically stable 3 years later

From January 2008 to December 2013, a total of nine patients with active advanced tuberculosis of the hip were treated by two-stage THA strategy, including five male subjects and four female subjects, with an average age of 50 years (range, 32–70 years). Each patient was diagnosed by detailed clinical, radiological, and laboratory evaluations before surgery and was confirmed by histological examination and biopsy culture postoperatively after the first operation. All the patients had significantly elevated erythrocyte sedimentation rate (ESR) and C-reactive protein (CRP) levels preoperatively. Out of the nine patients, four were found with sinus tracts to the thigh or pelvis (Figs. 2 and 3), and two of the four were observed with superinfection by bacterial culture from drainage liquid preoperatively. The hips of the other five patients were extensively destroyed by TB (Figs. 1 and 2). Clinical and radiographical features showed that all of these patients are conformed to Babhulkar’s standard of stage III and stage IV [2]. Details of the patients prior to operation are shown in Table 1.

**Table 1:** Details of the patients before the first operation

Patient	Medical co-morbidities	Pre-op ESR (mm/h)	Pre-op CRP (mg/L)	Sinus tract	Superinfection	Pre-op radiography	
						Chest X-ray	Hip X-ray/CT/MRI
1	None	58	46	−	−	HF	De,A,Sb
2	Hypertension	70	57	+	+, *S. aureus*	N	De,A,Sb
3	None	65	32	−	−	N	De,A
4	Pulmonary TB	68	43	+	−	AF	De,A,
5	None	40	25	−	−	N	De,A
6	None	55	28	−	−	N	De,A
7	DM	100	45	+	+, *S. aureus*	HF	De,A,Di
8	None	43	30	−	−	N	De,A
9	Pulmonary TB	80	33	+	−	AF	De,A

*N* normal, *HF* healed focus, *AF* active focus, *ESR* erythrocyte sedimentation rate, *CRP* C-reactive protein, *De* destruction of hip on both sides, *Sb* subluxation, *Di* dislocation, *A* abscess, *DM* diabetes mellitus

During the first stage, the inflamed soft tissues, necrotic bones, cold abscesses, and sinus tracts were debrided as thoroughly as possible prior to operation after at least 2 weeks of antituberculous therapy. Though there were no macroscopic inflamed tissues remained during operation, in view of the extensive destruction of the joint or the existence of sinus tracts, there might be always some residual focus. The prosthesis was not implanted immediately. The femoral heads of four patients were excised during the first operation due to serious destruction, and antibiotic-loaded cement spacers (7 g streptomycin and 1 g vancomycin per 40 g PMMA) [16, 17] were planted for them (Figs. 1 and 2).The femoral heads of the other five patients were relatively intact and were therefore reserved (Fig. 3). An epidural catheter was put into the joint during the operation in order to inject antituberculosis drugs into the hip postoperatively for all the patients. Specimens obtained in the operation were sent for pathological examination and biopsy culture. All patients were treated by antituberculous chemotherapy for a minimum of 3 months (two superinfection patients were also treated by intravenous culture-specific antibiotics for a minimum of 6 weeks) prior to the second operation. Management details during the first stage are shown in Table 2.

**Table 2:** Management during the first stage

Patient	Preoperative ATT (weeks)	Operative procedure	Postoperative ATT (months)	Antibiotic (days)	Postoperative investigate		ESR normal (months)	CRP normal (months)
					Biopsy culture	Histo-pathology		
1	2	Debridement	4	3	Negative	Ty	3	2
2	4	Debridement	5	42	M.TB	Ty	4	2
		Spacer			*S. aureus*			
3	3	Debridement	4	3	Negative	Ty	3	2
4	4	Debridement	5	5	M.TB	Ty	4	2
		Spacer						
5	3	Debridement	3	3	Negative	Ty	2	1
6	3	Debridement	3	3	Negative	Ty	2	1
7	4	Debridement	6	48	M.TB	ATy	5	3
		Spacer			*S. aureus*			
8	2	Debridement	4	3	Negative	Ty	3	2
9	4	Debridement	4	5	M.TB	Ty	3	2
		Spacer						

*ATT* antituberculous therapy, *M.TB* mycobacterium tuberculosis, *Ty* typical tuberculosis, *ATy* atypical tuberculosis

During the second stage (Table 3), if the antituberculous chemotherapy was effective and the tuberculosis was controlled, hip prosthesis could be implanted. For typical cases, the ESR and CRP were normal, the wound was healed well, the pain of the hip was relieved, and the body temperature was normal prior to the second operation. All THA, including seven cementless THA, one cemented THA and one hybrid THA, were performed using a posterior approach. The operation of two patients with segmental bone defect at the acetabulum used autograft. After the second operation, antituberculous chemotherapy was continued for 6–9 months, and the total duration of antituberculous chemotherapy was a minimum of 12 months after the first operation.

**Table 3:** Management during the second stage

Patient	Interval (months)	Operative procedure	Postoperative ATT (months)	Postoperative reinvestigate		ESR normal (months)	CRP normal (months)
				Biopsy culture	Histo-pathology		
1	4	UC-THA	8	Negative	Negative	3	1
2	5	UC-THA	7	Negative	Negative	4	2
3	4	UC-THA	8	Negative	Negative	3	1
4	5	H-THA	7	Negative	Negative	3	2
5	3	UC-THA	9	Negative	Negative	3	1
6	3	UC-THA	9	Negative	Negative	3	1
7	6	C-THA	9	Negative	Negative	5	3
8	4	UC-THA	8	Negative	Negative	3	1
9	4	UC-THA	8	Negative	Negative	3	2

*Interval* the time from the first operation, *ATT* antituberculous therapy, *UC-THA* uncemented THA, *H-THA* hybrid THA

All cases received systemic antituberculous chemotherapy simultaneously with local chemotherapy. The protocol of systemic antituberculous chemotherapy was as follows: isoniazid, rifampicin, ethambutol, and pyrazinamide were prescribed for the first 4 months; isoniazid, rifampicin, and pyrazinamide were prescribed for an additional 4 months; and isoniazid and rifampicin were prescribed for another 4 to 7 months. The local chemotherapy was only used after the first operation, and 0.1 g of isoniazid was injected into the hip through the epidural catheter once a day for 3–8 weeks or until the body temperature and ESR returned to normal. Then, the epidural catheter was removed. Periodic and regular blood tests were carried out to check the potential toxicity of these drugs, and the patients’ visual acuity was also examined regularly.

The patients were checked for follow-up once in every 4 weeks after the first operation until the second operation was carried out. After the second operation, clinical data were gathered once in every 4 weeks for the first 6 months. From the end of the sixth month to the end of 1 year, patients were specifically called for recheck once in every 8 weeks and once in every year thereafter. The functionality of the hip was evaluated by the Harris hip score (HHS) system. X-ray, ESR, CRP, and liver function test were included at every follow-up. Statistical analysis was performed using the SPSS version 17.0 for Windows. A matched *t* test was used to evaluate preoperative and postoperative ESR, CRP, and HSS; statistical significance was defined as *P* < 0.05.

### Ethical approval

This study was in compliance with the Helsinki Declaration and was conducted with approval from the Ethics Committee of Xiangya Hospital, Central South University. Written informed consents were obtained from all participants.

## Results

With an average follow-up of 40 months (range, 18–72 months), no reactivation of tuberculosis infection or superimposed infection was found in any of the patients. No patient demonstrated postoperative dislocation or neurological complications. The average HHS was increased from 35 (range 15–55) preoperatively to 91.5 (range 83–97) at the final follow-up. The ESR returned to normal (<15 mm/h) in a mean duration of 3.3 months after the second operation (range, 3–5 months). The CRP was returned to normal (<0.8 mg/dl) in a mean duration of 1.6 months after the second operation (range, 1–3 months). The X-ray film showed no prosthesis shift or loosening. The interval between the two operations is ranged from 3 to 6 months (average 4.2 months). No spacer dislocation or breakage was observed during this interval. The systemic antituberculous chemotherapy was performed for a total of minimum 12 months after the first operation. The local chemotherapy was used for 3–8 weeks through the epidural catheter after the first operation, and no intra- or postoperative catheter-related complications were detected. No liver injury was developed. One patient encountered DVT after the second operation and was cured with antithrombotic therapy. One patient with diabetes mellitus encountered incision-delayed healing and was cured with dressing change. The antituberculous chemotherapy was also prolonged to 15 months for this patient (Table 4).

**Table 4:** Follow-up

Patient	Follow-up (months)	Total postoperative ATT (months)	Reactivation	Harris hip score		Complications
				Preoperative (the first operation)	Postoperative (the second operation)	
1	72	12	No	30	93	No
2	60	12	No	28	94	No
3	45	12	No	40	90	No
4	30	12	No	34	88	DVT
5	18	12	No	30	95	No
6	24	12	No	40	89	No
7	48	15	No	15	83	Incision-delayed healing
8	33	12	No	43	94	No
9	30	12	No	55	97	No

*ATT* antituberculosis therapy, *DVT* deep venous thrombosis

## Discussion

Traditionally, the surgical treatment for advanced active TB of the hip includes debridement, arthrodesis, and resection arthroplasty combined with a certain period of antituberculosis therapy [2, 12]. Arthrodesis is no longer popular in the Asia-Pacific region because of the customary need for squatting, kneeling, and sitting cross-legged. With the advent of improved chemotherapy and the distinct disadvantages of arthrodesis among the above population, this treatment is now practiced infrequently [2]. Resection arthroplasty offers a painless and mobile hip, with removal of most of the infected tissues, and helps in eradicating the disease [18]. However, a significant reduction in the limb and instability persists [2, 19]. In addition, the conversion to THA after excision arthroplasty is complex and may be less satisfactory [19].

With the use of THA, hip tuberculosis in the quiescent stage has been successfully treated [1, 6, 7]. However, the reported time for the hip to become quiescent varies from 10 to 20 years [6, 7, 15, 20, 21]. Such a long waiting time would cause great dysfunction and seriously affect the life and work of patients.

Inspired by the success of spinal active tuberculosis treated with implant, several reports can be retrieved with respect to one-stage THA for patients with active tuberculosis [5, 8–11, 13, 14, 22], but it is a controversial treatment option due to the potential risk of reactivation of TB. Meanwhile, one-stage THA requires strict indications and proficient technical skills. Based on the summary of all reports, the success of one-stage THA for active TB of the hip is subject to three key points: (1) perioperative antituberculous therapy, (2) thorough debridement and complete curettage of infected tissues at the time of operation, and (3) best without sinus drainage [12].

Actually, according to our clinical experience, it is not easy to achieve thorough debridement for patients with advanced active TB of the hip because there is gross destruction of capsule, synovium, bones, and articular cartilage. The inflammatory and necrotic tissues and abscess are usually not restricted to the joint, but diffused to the periarticular area and even to the thigh or pelvis. Insect bites like cavities at the acetabulum and proximal femur can also cause trouble to curette them radically. In view of this situation, there are always some residual focuses. Shen et al. [10] and Yoon et al. [14] suggest that if thorough debridement cannot be achieved, a two-stage surgery should be considered.

As far as we know, this is the first paper focusing on two-stage total hip arthroplasty for patients with advanced active tuberculosis of the hip. With anti-TB medications, the two-stage THA protocol offers the greatest chance for the eradication of infection. This study reveals several advantages of the first-stage treatment. Firstly, it is conducive for antituberculosis drugs to exhibit a rapid effect when most necrotic, infected tissues and cold abscess are removed. In this study, the ESR and CRP of most patients returned to normal in 3 months after the first operation. Secondly, it can rapidly relieve the systemic symptoms of tuberculosis (such as fever) caused due to the above procedures. Thirdly, an epidural catheter was put into every hip during the operation, so that it was convenient to inject antituberculosis drugs into the hip postoperatively. Local chemotherapy can increase drug concentration, and it is beneficial to control the residual tubercular nidus [23]. Fourthly, the antibiotic-loaded cement spacer planted in the hip not only reserves the length and motion of the hip but also helps to eradicate infection [16, 17]. Lastly, specimens obtained in the operation for pathological examination and biopsy culture can help confirm the diagnosis. In this study, the ESR and CRP of all the patients were normal at the time of second operation, and intraoperative findings and pathological re-examination presented no active TB. To some extent, the prostheses can even be considered to be planted in the quiescent stage hips.

Most authors suggest that patients with sinus drainage are not well qualified for one-stage joint arthroplasty [3, 11, 12, 14, 15]. The presence of sinus drainage usually indicates pyogenic superinfections. Sinus tracts can also increase the difficulties of thorough debridement. Öztürkmen et al. [13] suppose that patients with infected sinus tracts extended into the pelvis or thigh are not suitable for one-stage THA because of the risk of reactivation due to the incomplete curettage and debridement of infected tissues. Yoon et al. [14] consider that patients with sinus tracts extended into the pelvis or thigh may not be a contraindication of primary THA for tuberculosis of hip, but they chose resection arthroplasty with a two-stage operation. Neogi et al. [8] reported that one patient with preoperative sinus drainage had encountered tuberculosis reactivation and superimposed infection through the non-healing sinus tract. There were four patients with sinus tracts to the thigh or pelvis in the present study, and two of them were detected with superinfection by bacterial culture preoperatively. The two-stage protocol was chosen for these four patients. Two patients with superinfection were also treated by intravenous culture-specific antibiotics for a minimum of 6 weeks after the first operation. None of them were found with reactivation of TB and pyogenic infection.

## Conclusions

Although one-stage THA is considered as a safe procedure for most patients with advanced active TB of the hip by some authors [24], our practical experience shows that two-stage THA is an alternative option to treat this challenging disease under some difficult conditions. The hip with sinus tracts or destroyed extensively with difficulties of thorough debridement at one operation may be indications of two-stage THA. With antituberculous medications, also antibiotic therapy for superinfection patients, the two-stage THA protocol offers the greatest chance for the eradication of infection. Of course, further studies with a large sample size and a longer follow-up are needed.
